# Lactic acid induces fibroblast growth factor 23 (FGF23) production in UMR106 osteoblast-like cells

**DOI:** 10.1007/s11010-021-04287-y

**Published:** 2021-11-03

**Authors:** Jana Alber, Michael Föller

**Affiliations:** Department of Physiology, University of Hohenheim, Garbenstraße 30, 70599 Stuttgart, Germany

**Keywords:** Phosphate, 1,25(OH)_2_D_3_, Klotho, Inflammation

## Abstract

Endocrine and paracrine fibroblast growth factor 23 (FGF23) is a protein predominantly produced by bone cells with strong impact on phosphate and vitamin D metabolism by targeting the kidney. Plasma FGF23 concentration early rises in kidney and cardiovascular diseases correlating with progression and outcome. Lactic acid is generated in anaerobic glycolysis. Lactic acidosis is the consequence of various physiological and pathological conditions and may be fatal. Since FGF23 production is stimulated by inflammation and lactic acid induces pro-inflammatory signaling, we investigated whether and how lactic acid influences FGF23. Experiments were performed in UMR106 osteoblast-like cells, *Fgf23* mRNA levels estimated from quantitative real-time polymerase chain reaction, and FGF23 protein determined by enzyme-linked immunosorbent assay. Lactic acid dose-dependently induced *Fgf23* gene expression and up-regulated FGF23 synthesis. Also, Na^+^-lactate as well as formic acid and acetic acid up-regulated *Fgf23*. The lactic acid effect was significantly attenuated by nuclear factor kappa-light-chain enhancer of activated B-cells (NFκB) inhibitors wogonin and withaferin A. Lactic acid induces FGF23 production, an effect at least in part mediated by NFκB. Lactic acidosis may, therefore, be paralleled by a surge in plasma FGF23.

## Introduction

Bone cells are the main source of fibroblast growth factor 23 (FGF23), a proteohormone with additional paracrine effects [1–4]. As an endocrine factor, it regulates vitamin D and phosphate homeostasis in the kidney by down-regulating *CYP27B1*, the key enzyme for activation of vitamin D, and NaPiIIa, the major Na^+^-dependent phosphate transporter [5–8]. In doing so, FGF23 inhibits the synthesis of 1,25(OH)_2_D_3_, active vitamin D [9], and enhances renal phosphate excretion resulting in lower plasma phosphate levels [10]. In the parathyroid glands, FGF23 decreases the secretion of parathyroid hormone (PTH) [11, 12]. Taken together, FGF23, 1,25(OH)_2_D_3_, and PTH are part of a complex hormone circuit influencing each other and controlling phosphate as well as Ca^2+^ homeostasis [5].

The aforementioned endocrine effects of FGF23 are dependent on a membrane receptor which assembles with transmembrane protein αKlotho [13–15]. Apart from being the co-receptor for FGF23, αKlotho has become known as a powerful anti-aging factor: Transmembrane αKlotho can release a fragment called soluble Klotho (sKL) with additional endocrine effects including anti-cancer activity [16–19]. FGF23 or αKlotho deficiency results in rapid aging and early onset of aging-associated diseases [14] whereas overexpression of αKlotho extends the life span of mice by about 30% [20].

Paracrine effects of FGF23 may affect the liver [21], heart [3, 22, 23], or immune system [24] and are, at least in part, αKlotho independent.

In clinical medicine, the plasma FGF23 concentration has been revealed as a valuable disease biomarker [25] which is positively correlated with progression and outcome in chronic kidney disease [26, 27] and further cardiovascular disorders [28–30].

Therefore, the regulation of FGF23 production is of high interest. Regulators of FGF23 include diet [31–33], PTH [34, 35], 1,25(OH)_2_D_3_ [36, 37], systemic factors such as inflammation [38–42], other hormones including erythropoietin (EPO) [43, 44] or insulin [45] as well as intracellular signaling pathways such as adenosine monophosphate-dependent kinase (AMPK) signaling [46].

Lactic acid is the result of anaerobic glycolysis. Its production is enhanced both under physiological conditions (e.g., physical activity above the anaerobic threshold leading to a marked surge in the plasma lactate concentration [47]) and pathological conditions (e.g., poorly controlled diabetes [48] or intoxication with metformin [49]). The resulting lactate acidosis [50] can have a wide spectrum of outcomes ranging from rapid recovery over life-threatening conditions [51] to death [52].

Since inflammation is a major driver of FGF23 production [53] and lactate induces pro-inflammatory activity [54], we sought to clarify whether and by which mechanism lactic acid regulates FGF23 production.

## Methods

### Cell culture

Cell culture experiments were conducted with UMR106 rat osteoblastic osteosarcoma cells (CRL-1661; ATCC, Manassas, VA, USA) cultured in Dulbecco’s Modified Eagle Medium (DMEM) high glucose (Gibco, Life Technologies, Darmstadt, Germany) supplemented with 10% fetal bovine serum (FBS) (Gibco, Life Technologies), 100 U/ml penicillin, and 100 μg/ml streptomycin (Gibco, Life Technologies) under standard culture conditions. Cells were pretreated with 10 nM 1,25(OH)_2_D_3_ (Tocris, Bristol, UK) for 24 h (6-well format; 2 × 10^5^ cells/well). Twenty-four hours later, they were treated with the indicated concentration of L-lactic acid or Sodium (Na^+^)-L-lactate (sodium chloride as vehicle control; Sigma–Aldrich, Schnelldorf, Germany; 24 h), nuclear factor kappa-light-chain enhancer of activated B-cells (NFκB) inhibitors withaferin A (Tocris; 500 nM, 24 h) or wogonin (Sigma; 100 µM, 24 h), or with vehicle only. Withaferin A and wogonin are potent inhibitors of NFκB signaling [55–57] that is a major enhancer of *Fgf23* gene expression [58]. In further series of experiments, UMR106 cells were treated with 22.8 mM formic acid (Carl Roth, Karlsruhe, Germany), 10 mM acetic acid (Carl Roth), or water for 24 h and, pH of supernatants was measured.

### Quantitative real-time PCR

Total RNA from UMR106 cells was extracted by means of RNA-Solv reagent (Omega Bio-Tek, Norcross, GA, USA). CDNA synthesis was performed with 1.2 µg RNA, random primers, and the GoScript™ Reverse Transcription System (Promega, Walldorf, Germany; 25 °C for 5 min, 42 °C for 1 h, and 70 °C for 15 min). *Fgf23* expression was determined by qRT-PCR on a CFX Connect™ Real-Time System (Bio-Rad, Feldkirchen, Germany) using GoTaq qPCR Master Mix (Promega). QRT-PCR conditions were 95 °C for 2 min, 40 cycles of 95 °C for 10 s, 57 °C for 30 s, and 72 °C for 25 s (2 μl cDNA, 0.25 μM (*Fgf23*) or 0.5 µM (*TATA box-binding protein, Tbp*) of each primer, 10 μl GoTaq® Green Master Mix (Promega) and RNAse-free water up to a total volume of 20 μl). *Fgf23* mRNA expression levels were referred to the expression levels of *Tbp*.

The following primers were used:

#### Rat *Fgf23*

- Forward (5ʹ → 3ʹ):TAGAGCCTATTCAGACACTTC.
- Reverse (3ʹ → 5ʹ): CATCAGGGCACTGTAGATAG.

#### Rat *Tbp*

- Forward (5ʹ → 3ʹ): ACTCCTGCCACACCAGCC.
- Reverse (3ʹ → 5ʹ): GGTCAAGTTTACAGCCAAGATTCA.

### ELISA

To determine FGF23 in the supernatant of UMR106 cells vivaspin 6 centrifugal concentrators (Sartorius, Göttingen, Germany) were used. *C*-terminal FGF23 was determined by ELISA (Immutopics, San Clemente, CA, USA) according to the manufacturer’s protocol. This ELISA exhibits a sensitivity of 4 pg/ml, an intra-assay precision coefficient of variation of 4.5–6.2%, and an inter-assay precision coefficient of variation of 4.4–5.9% according to the manufacturer. With regard to the binding region of the antibodies used, homology with rat amounts to 95% (capture antibody) and 90% (detection antibody) according to the manufacturer.

### Statistics

All data provided are arithmetic means ± standard error of mean (SEM), and *n* represents the number of independent experiments. Normality was examined with Shapiro–Wilk test. To determine statistical significance, data passing normality test were compared by paired t-test. For more than two groups, one-way analysis of variance (ANOVA) followed by Bonferroni correction was applied. Data that failed Bartlett’s test of homogeneity of variances were analyzed using Welch’s ANOVA test followed by Dunnett’s T3 correction. If Shapiro–Wilk showed *p* < 0.05 for comparison of more than two groups, nonparametric Kruskal–Wallis test with Dunn’s correction was used for statistical analysis. Test results with *p* < 0.05 were considered statistically significant. Statistical analysis was performed using GraphPad Prism 9 (Version 9.2.0; GraphPad Software Inc., San Diego, CA, USA).

## Results

### Lactic acid induces FGF23 production in UMR106 cells

**Fig. 1:**
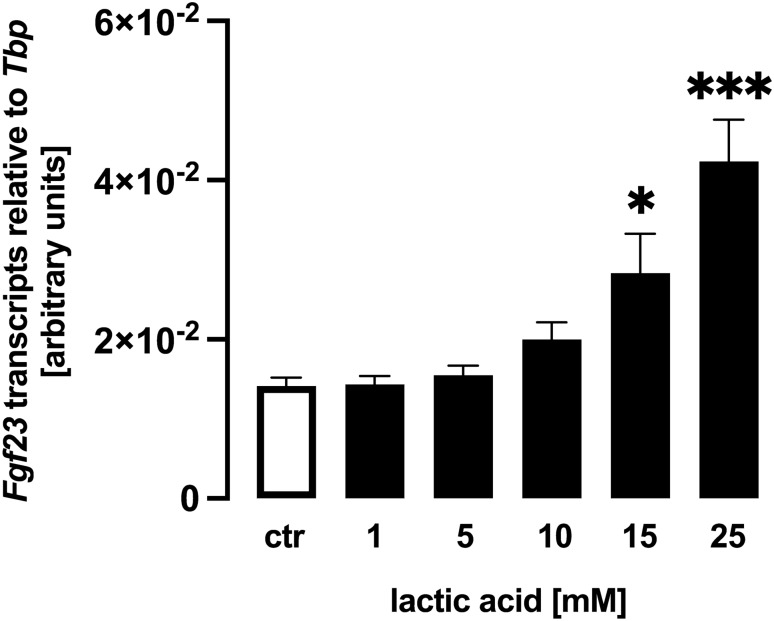
Lactic acid induces *fibroblast growth factor 23* (*Fgf23*) gene expression in UMR106 cells. Arithmetic means ± SEM (*n* = 6) of relative *Fgf23* mRNA abundance normalized to *TATA box-binding protein* (*Tbp*) expression in UMR106 cells incubated without or with lactic acid at the indicated concentration. ***p < 0.001; *p < 0.05 (Kruskal–Wallis test)

We utilized UMR106 osteoblast-like cells to study *Fgf23* gene expression and FGF23 protein production. In a first series of experiments, these cells were treated with different concentrations of lactic acid for 24 h, and subsequently *Fgf23* gene expression was determined by qRT-PCR. Lactic acid dose-dependently up-regulated the abundance of *Fgf23* mRNA (Fig. 1) pointing to a stimulation of *Fgf23* gene expression.

**Fig. 2:**
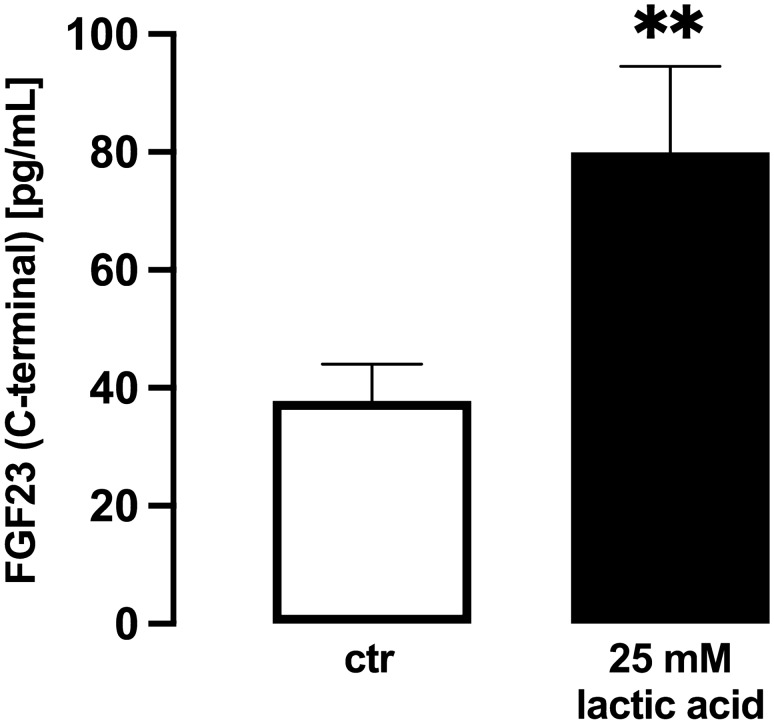
Lactic acid enhances FGF23 production in UMR106 osteoblast-like cells. Arithmetic means ± SEM (*n* = 6) of the *C*-terminal FGF23 protein concentration in the supernatant of UMR106 cells treated with or without 25 mM lactic acid for 24 h. **p < 0.01 (paired t-test)

Next, we aimed to study whether the stimulatory effect of lactic acid on *Fgf23* gene expression also translates into enhanced FGF23 protein secretion into the cell culture supernatant. To this end, we determined *C*-terminal FGF23 by ELISA. A 24 h treatment with 25 mM lactic acid significantly increased the concentration of C-terminal FGF23 in the cell culture supernatant of UMR106 cells (Fig. 2).

### Sodium lactate induces *Fgf23* expression in UMR106 cells

Lactic acid is a weak acid. We carried out pH measurements in the cell culture supernatant of UMR106 cells upon incubation without or with lactic acid or with other comparable weak acids, formic acid and acetic acid. As a result, a 24 h incubation of UMR106 cells without additional acid resulted in a supernatant pH of 7.44 ± 0.02 (*n* = 6), a value significantly different from the pH in the supernatant of cells incubated in the presence of 25 mM lactic acid (7.23 ± 0.04; *n* = 6; *p* < 0.001) or 22.8 mM formic acid (7.04 ± 0.03; *n* = 6; *p* < 0.001). In another series of experiments, a 24 h incubation without 10 mM acetic acid resulted in a supernatant pH of 7.45 ± 0.02 (*n* = 5), a value significantly different from the supernatant pH upon incubation with 10 mM acetic acid (7.37 ± 0.01; *n* = 5; *p* < 0.001). Since acidosis is a stimulator of FGF23 production [59, 60], we performed a new series of experiments to test whether the comparable pH-lowering effects of 25 mM lactic acid and 22.8 mM formic acid have similar effects on *Fgf23* gene expression. As a result, control cells had a relative *Fgf23* transcript abundance of 0.027 ± 0.001 (*n* = 9), a value significantly lower than in UMR106 cells incubated with 25 mM lactic acid (0.092 ± 0.005; *n* = 9; *p* < 0.05) or 22.8 mM formic acid (0.138 ± 0.012; *n* = 9; *p* < 0.001). In another series of experiments, a 24 h incubation of UMR106 cells with 10 mM acetic acid resulted in a relative *Fgf23* transcript abundance of 0.021 ± 0.001 (*n* = 5), a value significantly higher than in control cells (0.013 ± 0.000; *n* = 5; *p* < 0.001). Thus, acidosis is likely to be a major contributor to the stimulatory effect of lactic acid on FGF23.

**Fig. 3:**
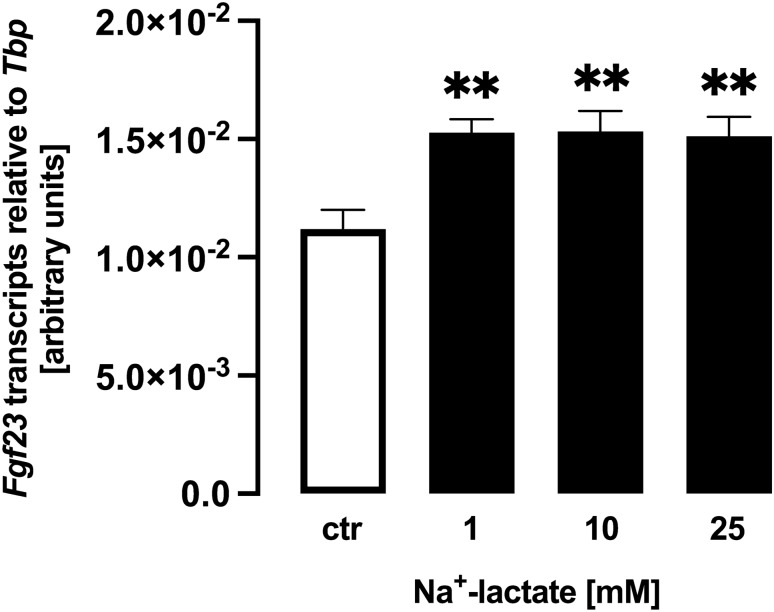
Na^+^-lactate induces *Fgf23* gene expression in UMR106 cells. Arithmetic means ± SEM (*n* = 5) of relative *Fgf23* mRNA abundance normalized to *Tbp* expression in UMR106 cells incubated without or with Na^+^-lactate at the indicated concentration. **p < 0.01 (one-way ANOVA)

With Na^+^-lactate, no acidosis can be induced. Therefore, we performed further experiments to clarify whether Na^+^-lactate impacts on *Fgf23*. Na^+^-lactate also up-regulated *Fgf23* gene expression in UMR106 cells within 24 h (Fig. 3), albeit to a lesser extent than lactic acid. Hence, lactate has the potential to stimulate *Fgf23* gene expression even without acidosis.

### Effect of lactic acid on *Fgf23* expression is blunted by withaferin A and wogonin

**Fig. 4:**
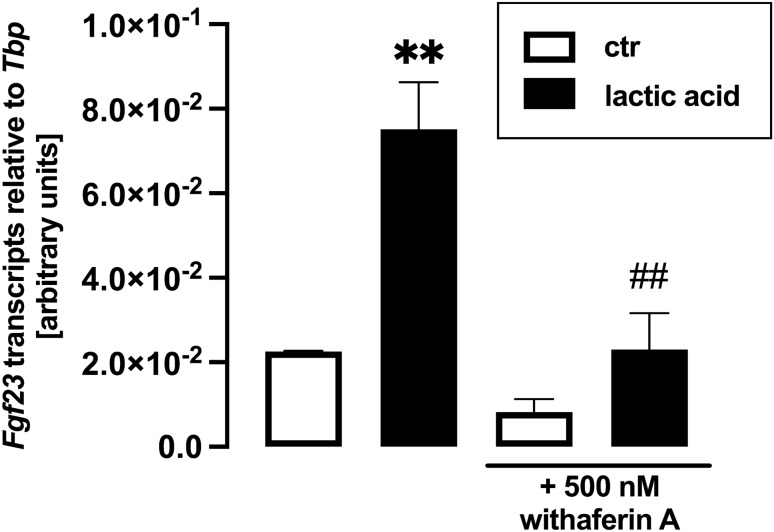
The effect of lactic acid on *Fgf23* gene expression is blunted by NFκB inhibitor withaferin A. Arithmetic mean ± SEM (*n* = 6) of relative *Fgf23* mRNA abundance normalized to *Tbp* expression in UMR106 cells incubated with or without 25 mM lactic acid in the presence or absence of 500 nM withaferin A for 24 h. **p < 0.01 indicates significant difference from the absence of lactic acid (control). ##*p* < 0.01 indicates significant difference from the absence of withaferin A. (Welch’s ANOVA)

**Fig. 5:**
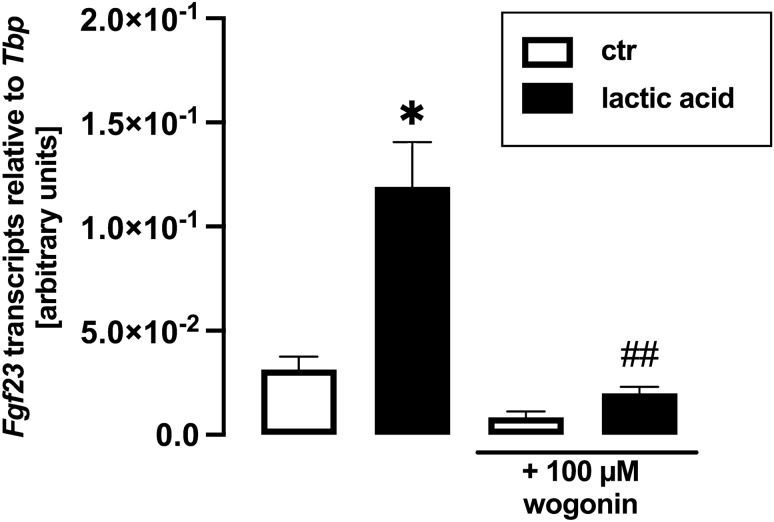
The effect of lactic acid on *Fgf23* gene expression is blunted by NFκB inhibitor wogonin. Arithmetic mean ± SEM (*n* = 9) of relative *Fgf23* mRNA abundance normalized to *Tbp* expression in UMR106 cells incubated with or without 25 mM lactic acid in the presence or absence of 100 µM wogonin for 24 h. *p < 0.05 indicates significant difference from the absence of lactic acid (control). ##*p* < 0.01 indicates significant difference from the absence of wogonin. (Kruskal–Wallis test)

Pro-inflammatory signaling mediated by transcription factor complex NFκB potently up-regulates FGF23 production [61], and lactic acid induces NFκB transcriptional activity [62]. Hence, we aimed to unravel whether NFκB is involved in the effect of lactic acid on FGF23. To this end, we treated UMR106 cells with and without lactic acid in the presence and absence of NFκB inhibitor withaferin A for 24 h. Withaferin A significantly blunted lactic acid-induced up-regulation of *Fgf23* gene expression (Fig. 4). The same held true for wogonin, another NFκB inhibitor (Fig. 5).

## Discussion

According to our study, lactic acid is a potent regulator of FGF23. This effect was, at least in part, mediated by NFκB. Lactic acid not only induced *Fgf23* gene expression in UMR106 osteoblast-like cells, but also C-terminal FGF23 protein secretion into the cell culture supernatant.

A major source of lactic acid is anaerobic glycolysis [63, 64]. Physical exercise stimulates anaerobic glycolysis and, hence, lactic acid formation in working muscle [64]. If the exercise remains below the anaerobic threshold, a steady state of lactic acid production in working muscle and utilization (e.g., in the liver for gluconeogenesis [65] or in the heart for energy production) exists with the lactic acid level remaining stable [66, 67]. The anaerobic threshold is in the range of 4–5 mM lactate [68, 69]. Physical activity above the anaerobic threshold cannot be sustained for longer time [47]. According to our results, concentrations of lactic acid and lactate above the anaerobic threshold triggered enhanced FGF23 production. In line with this, exercise has been shown to stimulate FGF23 production in mice [70], and it is tempting to speculate that lactic acid contributes to FGF23 production during physical activity. In humans, one study found an increase in plasma FGF23 of participants of Giro d’Italia (road bicycle race) – no lactate values are reported [71] – while another study did not find an impact of submaximal or high-intensity exercise on FGF23 [72] although the latter study found a moderate increase in lactate during high-intensity exercise. During strenuous exercise, plasma lactate is usually in a range below 10 mM [64] although peak values of 25 mM may be reached [73]. In our study, 15 mM lactic acid and 1 mM Na^+^-lactate were necessary to significantly up-regulate *Fgf23* gene expression. Definitely, further studies are needed to clarify whether physical exercise induces FGF23 through lactic acid in vivo.

A wide range of pathological conditions is associated with enhanced lactic acid formation causing lactic acidosis including uncontrolled diabetes mellitus [48] or, as a rare but dangerous adverse effect, metformin [49]. Lactic acidosis is a very serious condition as illustrated by a fatality rate of 25–50% in metformin-associated lactic acidosis [48, 49, 74]. In the latter case, the mean lactate concentrations may be 23 mM with some values as high as 35 mM [49, 75]. These concentrations are in the range of the highest lactic acid concentrations applied in our in vitro study. This supports the notion that lactic acid may be a relevant stimulator of FGF23 production also in vivo, at least in pathological lactic acidosis. As higher FGF23 levels are associated with poorer outcome in several disorders including kidney and cardiovascular diseases [27], higher FGF23 in severe lactic acidosis may also be indicative of a dismal prognosis. Moreover, severe acidosis worsens outcome in CKD [76] and higher FGF23 levels are associated with poorer outcome in this disorder [77]. Hence, normalizing plasma pH may also prove efficient in CKD due to the lowering of FGF23. Clearly, clinical studies are needed to address this question.

Acidosis is also a very common consequence of CKD [52]. Moreover, metformin-induced lactic acidosis typically affects patients with severe CKD [78]. Since FGF23 plasma levels go up early in CKD and predict prognosis [26, 79], lactic acid-induced FGF23 production may also be a mechanism relevant in CKD.

Addition of lactic acid caused a small but significant decrease in pH. Since acidosis has already been demonstrated to induce FGF23 production [59], we considered that the effect of lactic acid on FGF23 was, at least in part, due to acidosis. In line with this, formic acid or acetic acid induced a pH drop while stimulating *Fgf23* gene expression. However, also Na^+^-lactate, which is a weak base, was capable of enhancing *Fgf23*. Hence, cellular acidosis clearly contributes to lactic acid-induced FGF23 production, but may not fully explain it.

We could significantly blunt the stimulatory effect of lactic acid on FGF23 with two different inhibitors of NFκB, wogonin and withaferin A, pointing to an involvement of NFκB. In line with this, lactate is a stimulator of NFκB activity [62], and on the other hand, NFκB and inflammation have been demonstrated to be important inducers of FGF23 formation [39, 58].

## Conclusion

Taken together, our study demonstrates that lactic acid induces *Fgf23* gene expression and protein synthesis in vitro at concentrations encountered in vivo in lactic acidosis. This effect is, at least in part, mediated by NFκB and acidosis. High FGF23 concentrations in lactic acidosis may be suggestive for poor prognosis, although clinical studies are needed for clarification.

## Data Availability

The datasets used and/or analyzed during the current study available from the corresponding author on reasonable request.

## Abbreviations

1,25(OH)_2_D_3_: Calcitriol
AMPK: Adenosine monophosphate-dependent kinase
ANOVA: Analysis of variance
DMEM: Dulbecco’s modified eagle medium
ELISA: Enzyme-linked immunosorbent assay
EPO: Erythropoietin
FBS: Fetal bovine serum
FGF23: Fibroblast growth factor 23
NFκB: Nuclear factor kappa-light-chain enhancer of activated B-cells
PTH: Parathyroid hormone
qRT-PCR: Quantitative real-time polymerase chain reaction
sKL: Soluble klotho
SEM: Standard error of mean
Tbp: TATA box-binding protein
